# Antibiotic Synergy Interaction against Multidrug-Resistant *Pseudomonas aeruginosa* Isolated from an Abattoir Effluent Environment

**DOI:** 10.1100/2012/308034

**Published:** 2012-04-26

**Authors:** Etinosa O. Igbinosa, Emmanuel E. Odjadjare, Isoken H. Igbinosa, Phillips O. Orhue, May N. O. Omoigberale, Napoleon I. Amhanre

**Affiliations:** ^1^Department of Microbiology, Ambrose Alli University, Private Mail Bag 14, Ekpoma 310001, Edo State, Nigeria; ^2^Department of Microbiology and Biotechnology, Western Delta University, Private Mail Bag 10, Oghara 331100, Delta State, Nigeria; ^3^Department of Biochemistry and Microbiology, University of Fort Hare, Private Bag X1314, Alice 5700, South Africa

## Abstract

*Pseudomonas aeruginosa* is an opportunistic pathogen in environmental waters with a high prevalence of multidrug resistance. In this study the synergistic efficacy of synergy antibiotic combinations in multidrug-resistant *P. aeruginosa* strains isolated from an abattoir effluent was investigated. Water samples were processed using membrane filtration; *Pseudomonas* was isolated with Pseudomonas Isolation Agar and confirmed using polymerase chain reaction with specie-specific primer. Susceptibility studies and *in vitro* synergy interaction testing were carried out, employing agar dilution and Etest procedure, respectively. Resistance was noted for clinically relevant antipseudomonal agents tested. Finding from antibiotic synergy interaction studies revealed that cefepime, imipenem, and meropenem combined with amikacin resulted in statistically significant (*P* < 0.0001) *in vitro* antibiotics synergy interaction, indicating the possible use of this regimen in treatment of pseudomonal infections.

## 1. Introduction

The human opportunistic pathogen, *Pseudomonas aeruginosa*, is a major cause of infection-related mortality among the critically ill patients and carries the highest case fatality rate of all Gram-negative infections [1]. *Pseudomonas aeruginosa *is highly ubiquitous in water systems and has intrinsic antimicrobial resistance due to low outer membrane permeability, as well as an extensive efflux pump system [2, 3]. *P. aeruginosa* demonstrates resistance to multiple antibiotics, thereby rendering common antibiotic therapy ineffective [4]. The presence of multidrug-resistant *P. aeruginosa *in an aquatic milieu may be important for immune-suppressed or other at-risk individuals, for whom treatment difficulties have greater implications [5]. Some *P. aeruginosa *strains exhibit mutations in fluoroquinolone binding sites, the loss of porin channels, and increased beta-lactamase or cephalosporinase production [2, 3]. *P. aeruginosa* frequently acquires additional resistance mechanisms (plasmids) and routinely develops multidrug resistance throughout the course of a treatment regimen [3].

Sequential treatment for invasive infections has been typically considered as an option to improve results of monotherapy; however, combination therapy could be an alternative to monotherapy for patients with invasive infections that are difficult to treat, such as those due to multiresistant species and for those who fail to respond to standard treatment. Antimicrobial compounds used in combination might promote the effectiveness of each agent, with efficacy being achieved using a lower dose of each drug. Bantar et al. [6] studied the combination of amikacin with meropenem, ceftazidime, cefepime, and imipenem, respectively; results indicated that cefepime, especially in combination with amikacin, displayed bactericidal properties against carbapenem-resistant strains. The combinations with beta-lactams and an aminoglycoside or a fluoroquinolone remain a reasonable choice for treatment of invasive infections caused by *P. aeruginosa* [7].

Multidrug resistance in *P. aeruginosa* population is a pervasive and growing environmental problem, which is recognized as a threat to public health. Consequently, there is a need to conduct area-specific monitoring studies to profile different pathogens responsible for specific infections and their resistance patterns, so as to generate data that would help clinicians to choose the correct empirical treatment. In this study an attempt was made to investigate the antibiotics synergy pattern of *Pseudomonas aeruginosa* isolated from an abattoir in Benin-City, Nigeria.

## 2. Material and Methods

### 2.1. Samples Collection

The study was conducted in Benin-City, Nigeria between May and October, 2011. Water samples were collected on monthly basis from the abattoir effluent prior to discharge into the receiving water body, from 100 m downstream and upstream of the discharge point. Samples were collected in two liter (2 L) plastic containers that were previously sterilized with 70% (v/v) alcohol and rinsed with deionised water prior to usage. During sampling, sample containers were rinsed three times with sample water before filling with the sample. The actual samplings were done midstream by dipping each sample bottle at approximately 30 centimeter below the water surface, projecting the mouth of the container against the flow direction. After collection, the samples were protected from direct sunlight and transported in a cooler box containing ice packs to the laboratory for analyses. All samples were stored at 4°C and analyzed within 48 h of sample collection.

### 2.2. Bacterial Isolation

Water samples were analysed for the target bacterial pathogen using internationally accepted techniques and principles [8]. Prior to filtration, Samples were diluted 10-fold with sterile distilled water. Fifty milliliters (50 mL) of the appropriate dilution of each sample was filtered through a 0.45 *μ*m pore size membrane filter (Millipore), which was aseptically transferred to 45 mm Petri dishes with the appropriate selective media (Pseudomonas Isolation Agar) and incubated at 37°C for 48 h. After incubation, three to five randomly selected colonies with appropriate morphological characteristics were subcultured for purification using pseudomonas isolation agar plate which was incubated at 37°C for 24–48 h. The pure isolates were subjected to Gram staining and oxidase test. Only Gram-negative bacilli and oxidase-positive isolates were selected for biochemical reactions and using API 20 NE system. *Pseudomonas aeruginosa* (ATCC 27853) was used as control. The strips were then read, and final identification was made using API lab plus software (bio Merieux, Marcy l'Etoile, France).

### 2.3. Isolation of Genomic DNA

Single colonies of *P. aeruginosa *strains grown overnight at 37°C on Nutrient agar plates were picked, suspended in 100 *μ*L of sterile double distilled water and the cells were lysed using Heat Block for 15 min at 100°C. The cell debris was removed by centrifugation at 11,000 g for 2 min using a MiniSpin micro centrifuge and the supernatant used directly as template DNA or stored at −20°C until ready for use.

### 2.4. PCR Amplification Assay

Three to five isolates were obtained from Analytical Profile Index (API 20 NE system) identification of *P. aeruginosa *isolates and confirmed using polymerase chain reaction. All PCRs were performed in 22.5 *μ*L volume of reaction buffer containing 0.05 unit/mL Taq polymerase as recommended by the manufacturer (Fermentas Life Sciences) and 2.5 *μ*L of DNA template. Sterile double-distilled water was included in each PCR assay as a negative control, and positive controls contained DNA templates of *P. aeruginosa *ATCC 27853. All PCR was conducted using a MultiGene Thermal Cycler (Labnet International Inc., Edison, NJ, USA), at the following conditions: 95°C for 1 min; 40 cycles of denaturation at 95°C for 15 s, annealing at 58°C for 20 s; final extension at 68°C for 40 s, and holding temperature of 4°C. The primers used were: pa722F (5′-GGCGTGGGTGTGGAAGTC-3′) and pa899R (5′-TGGTGGCGATCTTGAACTTCTT-3′) amplicon size of 199 bp (Lutz and Lee, 2011). Electrophoresis of amplicons was performed with 1% agarose gel (Hispanagar, Spain) containing ethidium bromide (EtBr) 0.5 mg/L (Merck, SA) for 1 h at 100 V in 0.5 × TAE buffer (40 mM Tris-HCl, 20 mM Na-acetate, 1 mM EDTA, pH 8.5) and visualized under an UV transilluminator (BioDoc-It System, UVP Upland, Calif 91786, USA).

### 2.5. Agar Dilution Susceptibility Testing

Amikacin, aztreonam, cefepime, ciprofloxacin, piperacillin-tazobactam, ceftazidime, imipenem, and meropenem (all clinically relevant antimicrobial agents) were used to prepare antibiotic stock solutions as described by CLSI [9]. A 1 : 10 dilution was made of each antibiotic stock solution to be tested to obtain a final concentration of 2,560 *μ*g/mL. An agar dilution series (0.125-512 *μ*g/mL) was set up according to CLSI [9] procedure. Colonies of overnight culture on Mueller-Hinton agar medium were used to prepare and adjust inoculums as described by CLSI [9]. Two hundred microlitres (200 *μ*L) of each microbial suspension was placed into the wells of an inoculum. A growth control agar plate without antibiotics was inoculated first; thereafter all plates were inoculated starting with the lowest concentration. The inoculated spots were left to dry after which the inoculated agar plates were incubated at 37°C for 18 to 24 h.

### 2.6. The Minimum Inhibitory and Bactericidal Concentrations (MICs and MBCs)

The minimum inhibitory and bactericidal concentrations (MICs and MBCs) of amikacin, aztreonam, cefepime, ciprofloxacin, piperacillin-tazobactam, ceftazidime, imipenem, and meropenem were determined by broth microdilution method as described by CLSI, and CLSI criteria were used in the interpretation of the results [9]. Twofold serial dilutions, ranging from 0.125 to 256 *μ*g/mL, for each antibiotic were prepared in Mueller Hinton broth. The inoculum was prepared with 2-3 h broth culture of each isolate, adjusted to a turbidity equivalent to 0.5 McFarland Standard and diluted in Mueller Hinton broth to give a final concentration of 5 × 10^5^ cfu/mL. MIC was defined as the lowest concentration of antibiotic to completely inhibit visible growth. MBCs were determined by removing 10 *μ*L samples from each well, demonstrating no visible growth, and plated onto separate nutrient agar plates. After incubation at 37°C for 16–20 h, colonies were counted. MBC was defined as the lowest concentration of antibiotic to have at least 99.9% killing of the initial inoculum.

### 2.7. Etest Synergy Assay

Thirty-five multidrug-resistant isolates of *P. aeruginosa* were selected from agar dilution experiments. For the purpose of this experiment, multidrug resistance was defined as resistance to three or more test antibiotics. Antibiotics synergy studies using Etest strips were carried out and results interpreted as described by previous authors [10, 11]. Synergy was defined as a fractional inhibitory concentration (FIC) index of ≤0.5; indifference was indicated by FIC index >0.5 but ≤4, while antagonism was defined as a FIC index >4 [10]. Antibiotics included amikacin, aztreonam, piperacillin, piperacillin-tazobactam, cefepime, ceftazidime, imipenem, and meropenem. Bacterial colonies from overnight cultures were inoculated into sterile normal saline to obtain a 0.5 McFarland (optical density) standard. Muller Hinton agar plates were flooded with this suspension and left in a 37°C incubator to dry for 15 min. An amikacin Etest strip was applied to the dry plate and incubated at 35°C for 1 h. After 1 h the amikacin Etest strip was removed and a *β*-lactam Etest strip was placed exactly at the same position. Plates were incubated overnight at 37°C and read after 24 h.

### 2.8. Statistical Analysis

Susceptibility data were compared by using a Chi-square test with SPSS software for Windows, version 17.0. Both susceptibility and resistance were calculated as percentages with 95% confidence intervals. A *P* value <0.05 was considered to be statistically significant. 

## 3. Results

Among the total of 75 isolated presumptive *Pseudomonas aeruginosa*, by cultural and morphology characteristic, 55 were identified as *Pseudomonas aeruginosa* by the specie-specific primer employing PCR assay which was more sensitive for confirmation of the isolates. Minimum inhibitory concentration (MIC) and minimum bactericidal concentrations (MBCs) values of all isolates were determined (*n* = 55) (Table 1). The MBC values were generally equal or one to three times greater than those of MIC. All 55 isolates analyzed were resistant to more than three antipseudomonal agents. Thirty-five (*n* = 35) multidrug-resistant (MDR) strains were randomly selected for synergy testing using clinically relevant antibiotics included in the screening panel; there was MDR to amikacin, aztreonam, piperacillin, piperacillin-tazobactam, cefepime, ceftazidime, imipenem, and meropenem (Table 2). Two (5.71%) of the test isolates were sensitive to amikacin and aztreonam. Ten (28.57%) were sensitive to piperacillin; 4(11.42%) to piperacillin-tazobactam and cefepime; 7(20%) to ceftazidime; 3(8.57%) to imipenem and meropenem as shown in Table 3. Statistically significant indifference interaction was observed with aztreonam, piperacillin, and piperacillin-tazobactam in combination with amikacin *P* values 0.0018, 0.0012, and 0.0001, respectively. Cefepime, imipenem, and meropenem in combination with amikacin resulted in statistically significant *in vitro* antibiotics synergy interaction; *P* values 0.0001, <0.0001, and <0.0001, respectively. A combination of ceftazidime with amikacin resulted in significant antagonistic interaction *P* < 0.0001 (Table 3).

## 4. Discussion

*P. aeruginosa *is inherently resistant to many antimicrobial agents, mainly due to the synergy between multidrug efflux system or a type1 AmpC *β*-lactamase and low outer membrane permeability [12, 13]. The results of this study indicate that environmental *P. aeruginosa *isolated from an abattoir effluent has considerable levels of antibiotic resistance. Isolates demonstrated resistance to a wide range of clinically relevant antimicrobial agents, including amikacin, aztreonam, piperacillin, piperacillin-tazobactam, cefepime, ceftazidime, imipenem, and meropenem. Although *P. aeruginosa* is a model of antimicrobial resistance (due to a number of intrinsic factors), past research has found that the degree of resistance to antipseudomonal agents varies considerably. The current results are similar to past studies, which have found analogous prevalence levels of resistant *P. aeruginosa* in both the hospital and larger community [3, 14]. Although the levels of resistance among our test isolates were similar to those previously reported, it is important to draw the distinction between a nosocomial setting and the nonclinical, nonoutbreak, abattoir setting of the present study. In clinical situation, there is constant selective pressure to enhance the proliferation of multidrug-resistant strains. Known that *P. aeruginosa* has both intrinsic resistance and a dynamic ability to develop resistance during the course of infection, a high frequency of resistance is now expected in hospitals. However, in the nonclinical environments such as wastewater effluents and effluents from pharmaceutical industries, the presence of selective pressure has also increased antimicrobial resistance levels [15]. The presence of resistance of these environmental isolates to front-line antipseudomonal drugs may pose threat to the environment and to the receiving water body as these waste effluents are freely discharged into the environment.

In comparison with other previous studies [10, 11, 16], cefepime, imipenem, and meropenem in combination with amikacin exhibited remarkable synergy interaction (*P* = 0.0001 and *P* < 0.0001, resp.), and this could possibly be due to efflux activity particularly that of the multisubstrate efflux MexE-MexT-OprN [17], indicating the possible use of this procedure in treatment of pseudomonal infections. In light of the emerging resistance to carbapenems (imipenem and meropenem) and the limited utility of overcoming this resistance through prolonged infusion of high-dose carbapenem monotherapy, combination therapy may play a role in the treatment of infections associated with *P. aeruginosa* since it may provide the potential for synergistic effects between two different classes of anti-infectives [18]. Ceftazidime in combination with amikacin exhibited significant antagonistic interaction (*P* = 0.0001), making this combination an unlikely choice to treat invasive pseudomonal infections. However the antagonistic interaction with ceftazidime could be indicative of the presence of mutational derepression of Amp C type chromosomal *β*-lactamase or established integron-borne class A *β*-lactamase such as GES-2 [19, 20]. The presence of multidrug *P*. *aeruginosa* observed shows that this organism is a reality to deal with cautiously in the abattoir setting. Combination antimicrobial therapy with bactericidal activity is a common strategy often employed in an attempt to ensure reliable synergy or additive effects for the treatment of MDR *P. aeruginosa* infections and may reduce emergence of resistant strains during treatment. Antibiotic combinations including a *β*-lactam (cefepime), carbapenems (imipenem and meropenem), and an aminoglycoside (amikacin) have frequently produced an increased synergism effect *in vitro* in experimental models of aerobic Gram-negative bacillary infections. It has been suggested that such combinations are necessary in order to prevent the emergence of resistance during therapy. The synergistic effect could be as a result of weakening of cell wall or membrane components [21]. The findings of this study demonstrate the potential value and necessity of closely monitoring multidrug-resistant pathogens in an effluent environment given their public health significance.

## Figures and Tables

**Table 1:** Minimum inhibitory and bactericidal concentrations (MICs and MBCs) values of *Pseudomonas aeruginosa *strains.

Antibiotic	MIC (*μ*g/mL) (*n* = 55)	MBC (*μ*g/mL) (*n* = 55)
Amikacin	8–32	16–64
Aztreonam	8–32	16–64
Piperacillin	16–64	32–128
Piperacillin-tazobactam	16–32	32–64
Cefepime	4–8	8–32
Ceftazidime	1–8	2–16
Imipenem	2–32	4–64
Meropenem	1–8	2–16

**Table 2:** Multidrug-resistant (MDR) pattern of *P. aeruginosa. *

Isolate code	Multidrug-resistant profile
DPT14	AMI, AZT, MEM, PIP, CEF
DPT23	AZT, PIP, IMI, CEF
DPT18	AMI, PIPT, AZT, MEM
DPT40	AMI, AZT, CEFT, PIP
DPT25	AMI, IMI, MEM, AZT
DPT15	AZT, IMI, CET, PIP
DPT8	AMI, MEM, PIPT, IMI, PIP
DPT11	CEF, IMI, MEM, PIPT
UPST30	AMI, PIPT, AZT, MEM
UPST45	CEFT, AZT, PIP, AMI, CEF
UPST5	CEFT, IMI, AZT, PIPT
DWST31	AMI, CEFT, AZT, MEM
DWST51	AMI, AZT, MEM, PIP
DWST2	AMI, MEM, IMI, PIPT
DWST30	AMI, IMI, CEF, AZT
DWST18	IMI, AZT, CEFT, AMI
DWST34	IMI, AZT, CEFT, PIP CEF
DWST6	IMI, AMI, CEFT, AZT
DWST9	MEM, IMI, AMI, PIP, AZT, CEF
DWST16	MEM, IMI, PIP, CEFT
DWST37	MEM, IMI, AZT, CEF
DWST42	PIP, CEF, IMI, AMI
DWST28	AZT, IMI, PIPT, PIP
UPST35	AMI, PIPT, PIP, CEF
UPST39	AZT, IMI, AMI, CEF, CEFT
UPST27	IMI, AMI, AZT, MEM
AbSU5	MEM, AZT, CEF, CEFT
AbSU9	IMI, AMI, PIP, AZT, CEFT
AbSU36	AMI, AZT, CEF, CEFT
AbSU48	CEF, MEM, PIP, AMI
AbSU23	CEFT, IMI, PIPT, AZT
AbWT33	AMI, IMI, MEM, AZT
AbWT8	AZT, IMI, CEF, PIP
AbWT40	AMI, MEM, AZT, CEF
AbWT45	CEF, MEM, IMI, PIP

AMI: Amikacin; AZT: Aztreonam; PIP: Piperacillin; PIPT: Piperacillin-tazobactam; CEF: Cefepime; CEFT: Ceftazidime; IMI: Imipenem; MEM: Meropenem.

**Table 3:** Susceptibility and synergy interaction profile of multidrug-resistant *P. aeruginosa. *

Antimicrobial	% sensitivity to antibiotics only (*n* = 35)	Antibiotic in combination with amikacin (*n* = 35)		
		% synergy	% antagonism	% indifference
Amikacin	5.71	**—**	**—**	**—**
Aztreonam	5.71	10(0.0015)	35(0.3175)	65(0.0018)
Piperacillin	28.57	8(0.0025)	20(0.2754)	72(0.0012)
Piperacillin-tazobactam	11.42	16(0.4658)	8(0.8560)	76(0.0001)
Cefepime	11.42	82(0.0001)	1(0.3756)	17(0.0385)
Ceftazidime	20.00	8(0.0729)	67(0.0001)	25(0.5640)
Imipenem	8.57	74(<0.0001)	6(0.0867)	20(0.0265)
Meropenem	8.57	80(<0.0001)	4(0.0932)	16(0.0172)

Values in parenthesis represent (*P value*).
